# HDL Subclass Distribution Shifts with Increasing Central Adiposity

**DOI:** 10.1155/2019/2107178

**Published:** 2019-02-03

**Authors:** Nicholas J. Woudberg, Sandrine Lecour, Julia H. Goedecke

**Affiliations:** ^1^Hatter Institute for Cardiovascular Research in Africa, Department of Medicine, Faculty of Health Sciences, University of Cape Town, Cape Town, South Africa; ^2^Non-Communicable Diseases Research Unit, South African Medical Research Council, Parow Valley, Cape Town, South Africa; ^3^Division of Exercise Science and Sports Medicine, Department of Human Biology, University of Cape Town, Cape Town, South Africa

## Abstract

Although cross-sectional studies have shown that obesity is associated with lower concentrations of large high-density lipoprotein (HDL) subclasses, it is unknown if changes in HDL subclasses are related to changes in body fat and its distribution over time. We therefore assessed changes in HDL subclass distribution over a 5.5-year free-living follow-up period in 24 black South African women. At baseline and follow-up, body composition and body fat distribution were measured using anthropometry, dual X-ray absorptiometry, and computerized tomography. HDL subclass distribution was quantified using Lipoprint®. Over the 5.5-year follow-up period, body fat (+17.3 ± 4.5 kg, *p* < 0.05) and trunk fat mass (+7.4 ± 1.9%, % fat mass, FM, *p* < 0.05) increased, while leg fat mass (−2.53 ± 0.56%, % FM, *p* < 0.001) and the distribution of large (−6.43 ± 2.12%, *p* < 0.05) HDL subclasses decreased. A percentage decrease in large HDL subclasses was associated with a percentage increase in central fat mass (visceral adipose tissue (VAT) area, *p* < 0.05) and a percentage decrease in peripheral fat mass (leg fat mass). These preliminary findings suggest that a relative redistribution of body fat from the periphery to the abdominal region were associated with a decrease HDL subclass size in black South African women and provide a novel link between body fat distribution and lipidology in this population.

## 1. Introduction

The inverse relationship between cardiovascular disease (CVD) risk and high-density lipoprotein-cholesterol (HDL-C) levels has been well established in the literature [1]. However, clinical trials, aimed at raising HDL-C, have failed to reduce CVD risk [2]. Therefore, the trend has been to move away from using HDL-C as a strict marker of CVD risk in favour of measures of HDL functionality, composition, and distribution of the subclasses of HDL [3, 4]. Whilst there is significant debate regarding the relationship between specific HDL subclasses in relation to CVD risk, several epidemiological studies indicate that a lower proportion of larger HDL subclasses was associated with an increased risk for CVD [5–10]. However, the preferential associations of smaller HDL subclasses with cardioprotective and antioxidative components may reduce risk when enriched in patients, reviewed by [11].

In a study comparing black and white South African women with obesity, we found that higher BMI was associated with lower large HDL and higher intermediate HDL, and this finding was mainly driven by the association in white women with obesity [12]. Compared to white women, black South African women display a different body fat distribution, favouring higher abdominal and gluteofemoral subcutaneous adipose tissue (SAT) over visceral adipose tissue (VAT) accumulation [13].

Our previous work addressed whether free-living black South African women are susceptible to changes in body fat distribution over time and how this may impact on their cardio-metabolic risk. The women displayed a 9% increase in body fat and notably, the increase in fat mass was associated with a relative redistribution of body fat from the gluteofemoral region to the central region which, in turn, was associated with increased cardio-metabolic risk [14]. Interestingly, no changes in HDL-C concentrations were observed over the 5.5-year period [14]. This presented an interesting opportunity to test whether our previous findings regarding the association between HDL subclass and obesity may be casually related to changes in body fat or its distribution over time. Therefore, the aim of this pilot study was to measure the associations between changes in body fat and its distribution and changes in HDL subclass over a 5.5-year free-living follow-up period.

## 2. Materials and Methods

### 2.1. Study Population

The current pilot study comprises a subsample of the cohort originally described by Chantler et al. [14]. Briefly, women were recruited from an original sample of 240 premenopausal women tested between 2005 and 2006 [15]. The original cohort of 240 women was contacted and invited to participate in the longitudinal follow-up study (2010-2011). Of the original sample, 63 women were eligible to participate. Women were lost to follow-up due to illness (*n*=10), death (*n*=1), unwillingness to participate (*n*=38), and altered contact details (*n*=126). From this subsample of 63 women, 24 women were randomly selected for the current pilot study. The subsample did not differ in age or body composition from the original baseline sample [14]. The study was approved by the Research Ethics Committee of the Faculty of Health Sciences of the University of Cape Town (HREC REF: 101/2004).

### 2.2. Body Composition Assessment

At baseline and follow-up, weight, height, and waist (level of the umbilicus) circumference were measured. Whole body composition using dual-energy X-ray absorptiometry (Discovery-W®, software version 12.7.3.7; Hologic, Bedford, MA) was measured. *In vivo* precision was 0.7% and 1.67% for fat-free soft tissue mass and fat mass (FM), respectively. The distribution of body fat (arm, trunk, leg, android, and gynoid fat mass) was calculated as percentages of total fat mass (% FM). Computerized tomography, at the level of L4 and L5, was used to measure visceral adipose tissue (VAT) and subcutaneous adipose tissue (SAT) areas (Toshiba XpressHelical Scanner; Toshiba Medical Systems).

### 2.3. Lipid Profile Determination

Fasting blood samples were drawn for the determination of total cholesterol (total-C), triglycerides, high-density lipoprotein cholesterol (HDL-C), and low-density lipoprotein cholesterol (LDL-C) concentrations [14]. LDL-C was calculated using the Friedewald estimation [16].

### 2.4. Quantification of HDL Subclass Distribution

Serum HDL subclass was determined using the Lipoprint® HDL system (Quantimetrix, Redondo Beach, CA) [17, 18]. Briefly, serum (25 *µ*l) was mixed with Lipoprint loading gel (300 *µ*l), containing Sudan black dye which binds proportionally to the cholesterol present in the sample. The mix was placed onto the upper part of the high resolution 3% polyacrylamide gel. Photopolymerisation was carried out for 30 minutes at room temperature, and electrophoresis was performed for 50 minutes at 3 mA per gel tube. After a rest period of 30 minutes, gel tubes were scanned and analysed using the Lipoware software. The VLDL and LDL remained at the origin (retention factor (Rf) = 0.0) while albumin migrated as the leading front (Rf = 1.0). Between these, 10 HDL bands could be detected. HDL-1, HDL-2, and HDL-3 were defined as large HDL; HDL-4, HDL-5, HDL-6, and HDL-7 were defined as intermediate HDL and HDL-8, HDL-9, and HDL-10 were defined as small HDL. Each subclass was quantified and expressed as a percentage of total HDL.

### 2.5. Statistical Measures

Results are presented as mean ± standard error of mean (SEM) for normally distributed data and as median ± interquartile range (IQR) for non-normally distributed data. Non-normally distributed data were log transformed prior to statistical analysis and included VAT and triglycerides. Repeated measures analysis of variance was used to compare body composition, serum lipids, and HDL subclass distribution between baseline and follow-up. Pearson correlations were used to explore associations between changes in HDL subclass with changes in body fat and its distribution.

## 3. Results

### 3.1. Changes in Body Fat Distribution and Lipid Profile

At baseline, the mean age of participants was 29 ± 2 years, 73% were using hormonal contraceptives, and 71% were obese (BMI > 30 kg/m^2^). At follow-up, the use of contraceptives marginally increased to 77%, while all anthropometric and DXA-derived measures of body fatness increased (Table 1, *p* < 0.05), with 87.5% of participants gaining weight over the 5.5-year follow-up period. In addition, anthropometric and DXA-derived measures of central fat mass (waist and trunk fat) increased (*p* < 0.05), while lower body peripheral fat mass (leg and gynoid fat, as a percentage of total fat mass, % FM) decreased (*p* < 0.001). There was a tendency for VAT to increase (+35.6 ± 15.2, *p*=0.097), while arm and android fat mass (% FM), as well as SAT area did not change significantly over the follow-up period. Despite significant changes in fat mass and body fat distribution, HDL-C, total-C, and triglycerides did not change while LDL-C increased over the 5.5-year follow-up period (*p* < 0.05) (Table 2).

### 3.2. Changes in HDL Subclass

HDL subclass distribution changed over the 5.5-year follow-up period. There was a percentage decrease in the distribution of large (−14.4 ± 4.4%, *p* < 0.05), an increase in intermediate (+11.5 ± 3.2%, *p* < 0.005), and no change in small HDL subclasses (Table 2).

### 3.3. Association between Changes in HDL Subclasses and Body Composition

**Figure 1:**
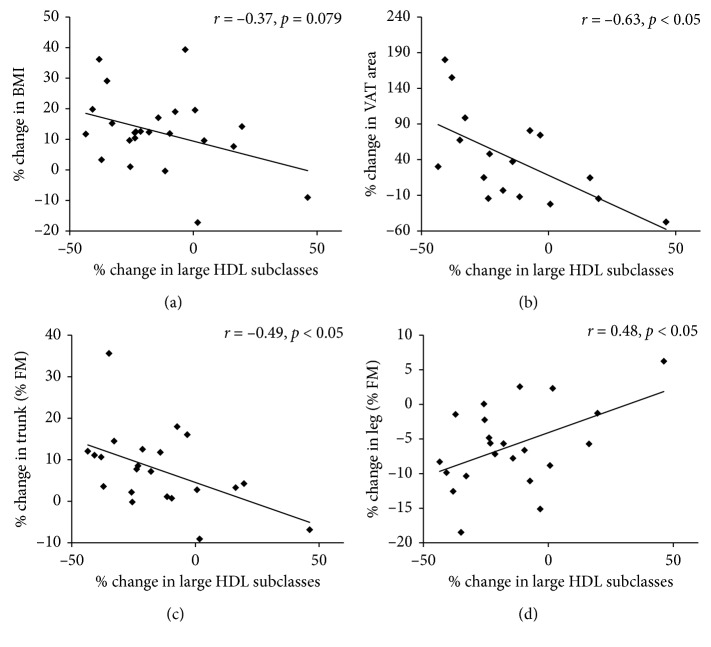
Associations between percentage changes in large HDL subclasses and body fat distribution. Percentage changes in BMI (a), VAT area (b), percentage trunk fat mass (c), and leg fat mass (d) were plotted against percentage changes in large HDL subclasses. Results represent Pearson correlation coefficients.

There was no significant association between percentage changes in BMI and large HDL subclasses (Figure 1(a)). However, a percentage increase in total body fat mass was associated with a decrease in large HDL subclasses (*r*=−0.47, *p* < 0.05). Moreover, the percentage decrease in large HDL subclasses was associated with a percentage increase in VAT area (*r*=−0.63, *p* < 0.05, Figure 1(b)) and trunk fat mass (% FM, *r*=−0.49, *p* < 0.05, Figure 1(c)). Percentage decreases in large HDL subclasses were also associated with percentage decreases in percentage leg fat (% FM, *r*=0.48, *p* < 0.05, Figure 1(d)).

## 4. Discussion

The novel finding of this pilot study was that, over the 5.5-year free-living period, an increase in total body fat and an increase in the centralization of body fat, characterized by an increase in VAT and trunk fat mass and a relative decrease in gluteofemoral fat (leg FM), were associated with a decrease in large HDL subclasses. Despite these changes in HDL subclass, HDL-C concentration remained unchanged. These findings are particularly novel in black African women, who are at heightened risk of CVD due to the increasing prevalence of obesity [19, 20].

In our previous study, we examined the association between obesity and HDL subclass distribution in a sample of normal-weight and obese black and white South African women [12]. We found that higher BMI was associated with lower large HDL subclasses, which has been reported in other cross-sectional studies [12, 21–23]. In the only other longitudinal study of this kind, a 5% gain in body weight over 6.5 years was associated with a decrease in larger HDL subclasses in a cohort from Finland [24]. Similarly, we showed that an increase in fat mass over 5.5 years corresponded to a decrease in large HDL subclasses in black South African women. Notably, we showed, for the first time to our knowledge, that the decrease in large HDL subclasses was associated with an increase in the centralization of body fat. This association was specific to increases in VAT and trunk fat mass and the relative decrease in gluteofemoral fat.

Centralization of body fat was previously shown in this population as a predictor of insulin resistance and raised triglyceride concentrations, but was not associated with HDL-C concentrations [14]. Cross-sectional data demonstrated that higher abdominal fat and VAT have previously been shown to be related with lower HDL particle size [25–27]. In contrast, changes in the distribution of VAT following an exercise intervention were not associated with changes in lipoprotein size [28]. VAT, due to its high lipolytic activity, increases mobilization of fatty acids, which are released directly into the hepatic portal system [29]. In contrast, peripheral SAT acts as a metabolic sink to sequester excess fatty acids [30]. The higher fatty acid flux, in addition to the proinflammatory nature of VAT, may then present a causal relationship between increased VAT and decreased HDL subclass size. Large cohort studies have shown that decreases in large HDL subclasses were associated with increased risk of CVD [5–7]. In support of our findings, cross-sectional analysis of women with central obesity demonstrated negative associations between VAT and HDL subclass size [21, 31]. This study therefore provides the first evidence how changes in adiposity in a black African population are associated with changes in HDL subclass.

Despite a low sample number, the study was capable of showing significant associations between measurements of HDL subclass and body composition in sample black African women. While the low sample number does limit the conclusions of this pilot study, however, the study does provide a preliminary understanding of how body composition changes may influence changes in lipidology. Whilst a previous study showed that, in the larger population group, dietary changes were not associated with changes in body composition, the variability with questionnaire-based assessments prevented the inclusion of these data in the pilot study [32].

Following the findings of this pilot study, future studies should seek to examine the links between body composition and measures of lipoprotein subclass in larger populations, including both men and women. In addition, it will be worth considering how levels of HDL associated apolipoprotein A1 and associated enzyme levels (paraoxonase) may be altered over time. Further, measures of inflammatory stress such as C-reactive protein would be helpful to delineate the effect of changes in inflammation with weight gain on these relationships.

## 5. Conclusions

For the first time, we have shown that, in women, centralization of body fat is associated with decreases in large HDL subclasses, which have consequences for increased cardio-metabolic and CVD risk. Critically, changes in body composition were not associated with changes in HDL-C, which is traditionally measured as a CVD risk factor. This study therefore provides novel evidence in an African setting of how weight gain and changes in body fat distribution may alter lipid biochemistry by changing HDL subclass distribution. This creates the potential for future, larger cohort studies, to examine the long-term predictive capacity of HDL subclass in determining the risk for cardiometabolic and CVD.

## Figures and Tables

**Table 1:** Changes in body composition and body fat distribution.

	Baseline	Follow-up	% change	*p* value	*n*
Age (yrs)	29 ± 2	35 ± 2	+20 ± 1	<0.001	24
*Body composition*					
Weight (kg)	85.6 ± 3.5	94.6 ± 3.4	+11.8 ± 2.5	<0.001	24
BMI (kg/m^2^)	33.3 ± 1.4	36.8 ± 1.5	+12.4 ± 2.5	<0.001	24
Waist circumference (cm)	98 ± 3	110 ± 3	+12.5 ± 2.1	<0.001	24
Body fat (kg)	36.6 ± 2.6	42.2 ± 2.5	+17.3 ± 4.5	0.001	22
Body fat (%)	42.4 ± 1.4	45.3 ± 1.2	+6.4 ± 2.2	0.005	22
Arm fat (% FM)	11.6 ± 0.4	11.6 ± 0.4	−0.2 ± 2.4	0.802	22
Trunk fat (% FM)	42.6 ± 1.1	45.8 ± 1.0	+7.4 ± 1.9	<0.001	22
Leg fat (% FM)	43.0 ± 1.3	40.2 ± 1.2	−5.8 ± 1.2	<0.001	22
Android fat mass (% FM)	7.7 ± 0.3	8.1 ± 0.3	+5.7 ± 3.8	0.148	22
Gynoid fat mass (% FM)	19.0 ± 0.5	18.1 ± 0.5	−4.3 ± 1.1	<0.001	22
SAT area (cm^2^)	494 ± 38	505 ± 35	+12.3 ± 6.8	0.122	17
VAT area (cm^2^)	54.5 ± 55.2	71.7 ± 72.8	+35.6 ± 15.2	0.097	17

Results represent mean ± SEM. For VAT area, results are expressed as median ± IQR. Arm, trunk, leg, android, and gynoid fat mass are expressed as percentages of total fat mass. Unadjusted *p* values are tested for significance of the time. VAT, visceral adipose tissue; SAT, subcutaneous adipose tissue.

**Table 2:** Changes in serum lipids and HDL subclass distribution.

	Baseline	Follow-up	% change	*p* value	*n*
*Serum lipid*s					
Total-C (mmol/L)	3.9 ± 0.2	4.2 ± 0.2	+9.5 ± 3.8	0.058	24
LDL-C (mmol/L)	2.2 ± 0.1	2.5 ± 0.1	+16.6 ± 5.6	0.033	24
HDL-C (mmol/L)	1.3 ± 0.1	1.3 ± 0.1	+1.6 ± 3.9	0.823	24
Triglycerides (mmol/L)	0.60 ± 0.50	0.85 ± 0.68	+37.4 ± 18.7	0.154	24
*HDL subclass*					
Large (%)	36.6 ± 1.7	30.8 ± 2.3	−14.4 ± 4.4	0.002	24
Intermediate (%)	48.1 ± 1.0	53.3 ± 1.0	+11.5 ± 3.2	<0.001	24
Small (%)	15.2 ± 1.2	15.9 ± 1.3	+12.2 ± 11.1	0.638	24

Results are expressed as mean ± SEM. For triglycerides, results are expressed as median ± IQR. Unadjusted *p* values are tested for significance of the time.

## Data Availability

The data used to support the findings of this study are available from the corresponding author upon request.
